# Epigenetic Diversity Underlying Seasonal and Annual Variations in Brown Planthopper (BPH) Populations as Revealed by Methylation- sensitive Restriction Assay

**DOI:** 10.2174/0113892029276542231205065843

**Published:** 2023-12-28

**Authors:** Ayushi Gupta, Suresh Nair

**Affiliations:** 1 Plant-Insect Interaction Group, International Centre for Genetic Engineering and Biotechnology (ICGEB), Aruna Asaf Ali Marg, New Delhi, 110067, India;; 2 Current Address: Institute of Molecular Plant Sciences, University of Edinburgh, Edinburgh EH 93BF, UK

**Keywords:** Phenotypic plasticity, seasonal polymorphisms, methyltransferases, epialleles, *Nilaparvata lugens*, brown planthopper

## Abstract

**Background:**

The brown planthopper (BPH) is a monophagous sap-sucking insect pest of rice that is responsible for massive yield loss. BPH populations, even when genetically homogenous, can display a vast range of phenotypes, and the development of effective pest-management strategies requires a good understanding of what generates this phenotypic variation. One potential source could be epigenetic differences.

**Methods:**

With this premise, we explored epigenetic diversity, structure and differentiation in field populations of BPH collected across the rice-growing seasons over a period of two consecutive years. Using a modified methylation-sensitive restriction assay (MSRA) and CpG island amplification-representational difference analysis, site-specific cytosine methylation of five stress-responsive genes (*CYP6AY1, CYP6ER1, Carboxylesterase, Endoglucanase, Tf2-transposon*) was estimated, for identifying methylation-based epiallelic markers and epigenetic variation across BPH populations.

**Results:**

Using a cost-effective and rapid protocol, our study, for the first time, revealed the epigenetic component of phenotypic variations in the wild populations of BPH. Besides, results showed that morphologically indistinguishable populations of BPH can be epigenetically distinct.

**Conclusion:**

Screening field-collected BPH populations revealed the presence of previously unreported epigenetic polymorphisms and provided a platform for future studies aimed at investigating their significance for BPH. Furthermore, these findings can form the basis for understanding the contribution(s) of DNA methylation in providing phenotypic plasticity to BPH.

## 1 INTRODUCTION

The brown planthopper (BPH; *Nilaparvata lugens*) is one of the most damaging and devastating pests of rice and is responsible for huge economic losses to farmers [1]. It is a small (2-3.5 mm in body length), brownish, monophagous sap-sucking (phloem-feeding) insect that exclusively feeds on rice and has become a major threat to global rice production. The life cycle of the BPH comprises of an egg (laid on either side of the midrib of the leaf sheath of rice plants), which hatches into a nymph that eventually matures into an adult [2]. BPH adults exhibit density-dependent wing dimorphism with macropterous (long-winged) and brachypterous (short-winged) forms. Macropterous insects have the ability to migrate over long distances. Furthermore, owing to its migratory nature, shorter life cycle, high reproductive rate, and remarkable stress resilience, BPH has invaded almost all rice-growing ecosystems. Currently, the use of pesticides appears to be the most convenient option for its immediate control. However, pesticides not only add to the production costs but also negatively impact the environment. Moreover, the indiscriminate use of broad-range chemical pesticides seriously threatens human health [3]. In addition, their excessive use imposes selection pressure on insect populations, thereby leading to the emergence of highly virulent and resistant strains, which further compounds the problem of pest management [4]. Besides, “pesticide breakdown” appears to be an evolutionary inevitability, especially for migratory pests like BPH [5]. Collectively, all these factors lead to the necessity of developing effective management strategies that not only aim at reducing the ecological fitness of BPH and delaying the evolution of resistant/virulent strains but also take into account the interests of farmers, society, and the environment. But before we embark on identifying and developing suitable and, more importantly, effective pest-management strategies, we must possess adequate information on the pre-existing genetic and phenotypic variation within BPH populations, which are important determinants of their ecological distribution, evolution, and capacity to adapt to environmental fluctuations.

Being highly migratory, BPH populations often invade and colonise different agro-climatic zones of the world [6]. Their capacity to rapidly adapt to different environments and ability to tolerate various biotic and abiotic stresses indicate the existence of physiological, biochemical and/or genetic plasticity within BPH populations, which, under certain circumstances, allows some individuals to survive better and outperform other members of the population and thereby, leading to adaptation [7]. Previous studies have demonstrated that BPH in the subtropical and temperate regions with almost round-the-year rice cultivation shows seasonal variations, which is linked to the region’s cropping pattern(s) [8, 9]. These changes are induced by changes in light intensity and temperature across seasons, which influence BPH flight and form (Brachypterous (short-winged) or Macropterous (long-winged)). Additionally, Satpathi *et al.* [10] and Claridge *et al.* [11] have shown that environmental variations lead to changes in food consumption and utilisation rates, fecundity and reproductive rate, mean generation length and population doubling time. These observations have been reinforced by data obtained from several other studies on BPH populations found in the tropical and temperate regions of Asia [12-15]. However, the molecular basis for these variations observed in BPH populations remains largely unexplored.

Phenotypic variation in insect populations not only serves as the basis for adaptation and selection but is also central to its ecology and evolution [16]. Usually, phenotypic alterations result from morphological, biochemical, or behavioural changes at the individual level in response to specific environmental cues [17]. Such alterations can cause substantial changes in the developmental trajectory and/or modifications in an insect's phenotypic state or activity (*e.g*., metabolism) under hostile environments – all of which can contribute to its remarkable resilience to stress [16, 18]. Although such variations are essentially an outcome of the inherent genetic variations, it is also, to some extent, influenced by external environmental factors [19]. Generally, it is observed that a genetically homogenous insect population can be phenotypically diverse due to the differences in climate, dietary resources and other external environmental factors [16]. While on the one hand, BPH shows highly efficient and rapid strategies to adapt to fluctuating environmental conditions (abiotic as well as biotic), on the other hand, it is difficult to gauge how such a wide array of responses is possible considering that BPH individuals lack sufficient genetic diversity within BPH populations [20], to affect such diverse adaptations. Therefore, it seems reasonable to believe that other mechanisms are likely contributing to its fitness and adaptability. In this regard, our earlier study has already reported and indicated that seasonal shifts in BPH microbiome structure and composition underlie its adaptation and survivability across changing climatic conditions [21]. However, such phenotypic changes in BPH populations across seasons are also possible through the action of epigenetic processes, such as DNA methylation, which, without changing the DNA sequence, can result in altered insect phenotypes as a response to external environmental stimulus/stimuli [22, 23].

Considering that virulence in BPH is under polygenic control [24] and mechanisms, such as DNA methylation, can potentially induce or suppress the activity of these genes, variation in DNA methylation patterns can alter the phenotype and contribute towards the rapid adaptive response(s) of BPH. DNA methylation as a response to environmental cues involves the covalent addition of a methyl group to the 5-carbon position of a cytosine residue resulting in the formation of 5-methylcytosine (5mC) in a reaction catalysed by DNA methyltransferase (DNMT) enzymes [25]. This phenomenon is highly conserved in eukaryotes; however, unlike higher organisms with high levels of DNA methylation, insects generally display low levels of methylation ranging from 0-14% [26]. BPH belongs to the order Hemiptera – an order that is represented by insect species with the second-highest levels of DNA methylation across all insect orders studied thus far [27]. Besides, it has been reported that planthoppers possess a functional DNA methylation machinery with all of its three DNA methyltransferase (*DNMT1*, *DNMT2*, *DNMT3*) genes active [28-30]. In addition, our earlier studies have already shown the role of demethylation/methylation in conferring adaptive advantages to BPH [9, 31]. However, to what extent BPH populations undergo epigenetic changes with changing environmental conditions across seasons is still unclear.

While genetic variation is mainly responsible for most of the heritable phenotypic variations, a part of this can also be an outcome of epigenetic influence [9, 32-35]. Currently, there is no available information in this regard. Therefore, we deemed it pertinent to investigate the levels of epigenetic diversity between and within BPH populations, which could account for their varied virulence and phenotypes. We believe that understanding and deciphering these epigenetic variations present within BPH is crucial for our understanding of BPH populations, survival strategies, and their capacity to respond to hostile environments. Besides, the information obtained from such studies could also be exploited to reliably distinguish populations of BPH, which, currently, is not consistently possible using conventional molecular biology tools. Moreover, owing to the continuous and rapid emergence of highly virulent/resistant BPH strains, the differentiation of populations at the intraspecific level is crucial for understanding biodiversity, speciation and adaptive changes in BPH. Additionally, the information regarding these mechanisms could also be utilised to develop effective management strategies for this economically important pest of rice.

With this view, the present study attempts to assess and identify environmentally-induced epigenetic variations within and between the BPH populations found during the different rice-growing seasons. To establish the presence of intra- and inter-population variations in DNA methylation, we used a modified methylation-sensitive restriction assay (MSRA [36-40];), coupled with CpG island amplification-representational difference analysis, as a tool for screening the site-specific cytosine methylation of CpG islands within five selected stress-responsive genes, in the BPH populations collected over different rice growing seasons. The CpG sites analysed for methylation/demethylation in the present study have been previously shown to be associated with the BPH’s rapid adaptive nature to pesticide and nutritional stresses [9]. In addition, it has also been demonstrated that the demethylation of CpG sites (corresponding to the five stress-responsive genes analysed in the present study) affects BPH survival under hostile environments [9].

The technique employed in the present study enables rapid detection of the methylation status of any genomic region prone to methylation. We successfully implemented this technique in our study for the rapid and cost-effective identification of methylation-based epiallelic markers contributing to adaptive epigenetic variation in the wild populations of BPH. In addition, the insights thus obtained with regard to methylation as an additional source of variation, along with the methylation status of these genes, are discussed in relation to its influence on insect adaptation and how such adaptations are integral to deciphering the response(s) of BPH populations to changing climatic conditions. We believe it is crucial to understand the link between seasonal weather systems and patterns and population fluctuations of this highly migratory insect pest of rice so as to anticipate likely variations in BPH populations and, subsequently, use this information to devise and deploy appropriate pest management strategy(ies). Further, when extended to other insect models, the information obtained from this study can form the basis for establishing a correlation between methylation-based epigenetic variation and adaptive genetic diversity in insect populations.

## 2 MATERIALS AND METHODS

### 2.1 BPH Populations

In the Northern regions of India, rice is mainly sown during May–June and harvested during October–November. Therefore, to elucidate epigenetic diversity within and among BPH populations, BPH insects found in Delhi (28.52° N, 77.16° E) during the rice-growing seasons (in June, August and November), collected for two consecutive years (2017-2018), were analysed. Here, it is worth mentioning that the external climatic conditions (*e.g*., temperature, rainfall, and humidity) vary significantly across these months. For instance, while the average temperature, humidity and rainfall during the month of June are 38°C, 80% and 55%, respectively, it comes down to 20°C, 25% and 2% in November, and these changes can drastically impact the BPH epigenome. Moreover, BPH is generally not found in Delhi during winter months (*i.e*., January till April) as the Northern parts of India experience harsh winters with temperatures dipping to less than 8°C, which is lethal for BPH [21]. Therefore, we analysed BPH populations found in Delhi during the peak rice growing season (*i.e*., in June, August and November). The rice plants were mostly in the tillering or in the grain-filling stage when the BPH individuals were collected. These insects collected at different time points (across the rice growing season in Delhi) constituted the six populations of BPH (*i.e*., June-17’ and June-18’, Aug-17’ and Aug-18’, Nov-17’ and Nov-18’ collected insects) analysed in the present study. The naturally occurring BPH individuals (~100/population) feeding on rice plants were randomly collected from the same rice fields (where the field management practices, including fertilization, pesticide (imidacloprid and a mixture of profenophos and cypermethrin) spraying and weeding, were routinely followed) within the same locality (during both 2017 and 2018) and stored in 100% ethanol at -20°C till further use. Our previous studies have shown that BPH exhibits huge epigenetic diversity across its life stages [9]. Therefore, to negate the effect of differences in the methylation status due to different life stages, we restricted our analysis to the BPH adults.

### 2.2 Genomic DNA Extraction and Quantification

As the primary objective of this study was to establish the presence of intra- and inter-population variations with regard to DNA methylation, genomic DNA was extracted from the whole body of the eight BPH individuals of each population using the tissue DNA extraction kit (Vivantis, Malaysia), as per the manufacturer’s instructions. DNA was quantified on the NanoDrop Spectrophotometer (Thermo Fisher Scientific, USA), and the quality was assessed by gel electrophoresis (0.8% TBE agarose gel) [41]. Next, genomic DNA from four BPH individuals of a population were pooled in equal amounts. Each pool representing a population contained DNA from 1 macropterous male, 1 macropterous female, 1 brachypterous male and 1 brachypterous female. All subsequent analyses were carried out on pooled DNA samples, representing each population in duplicate. This allowed the determination of the environmentally-induced epigenetic variations vis-à-vis their DNA methylation patterns between and within BPH populations, irrespective of their form, sex and lineage.

### 2.3 Shortlisting of Genes and CpG Island Prediction

The genes shortlisted for screening in this study were *CYP6AY1 (AY1), CYP6ER1 (ER1), Carboxylesterase (Est), Endoglucanase (EG) and Tf2-transposon (Tf2).* These genes were selected based on their functional relevance and involvement in BPH survival and fitness and their potential role in mediating rapid adaptations in BPH (Table **1**). Further, as previously shown that BPH has very low levels of methylation, mostly confined to the CpG islands across insect orders [26], we restricted our analysis to the predicted CpG islands within these genes. Therefore, the nucleotide sequence (CpG island) that is prone to methylation, owing to its unusually high GC content and a higher frequency of CpG dinucleotides, was predicted across the selected genes using a web-based tool, EMBOSS CpGplot. The criteria for the identification of putative islands were as follows: the ratio of observed CpG to expected CpG (Obs/Exp value > 0.6), sequence length (> 200 bp) and the GC content (> 50%). Subsequently, the nucleotide sequences of the predicted CpG islands of all the selected genes were retrieved and screened for methylation-sensitive and insensitive restriction enzyme sites using the MacVector (MacVector Inc., USA; version 15.5). The restriction sites were chosen based on their established role and functional relevance in mediating BPH survival under stress [9]. DNA methylation status is often under or over-represented by the underlying variation in DNA sequence. This can be a serious problem, especially while targeting the populations of highly migratory insects like BPH, and needs to be tackled by an appropriate experimental design. Therefore, to obtain reliable information regarding DNA methylation in BPH, we restricted our analysis to the CpG sites conserved across BPH populations.

### 2.4 Restriction Digestion

The list of 5 mC sensitive restriction enzymes was obtained from the gold standard REBASE resource (http://rebase.neb.com/rebase/rebase.html). For the current study, the restriction enzymes were selected based on the following criteria: (1) the presence of at least one restriction site within the identified CpG islands, and (2) possess a restriction site for an isoschizomer that is insensitive to methylation but recognizes the same position within the restriction site. Based on these criteria, three pairs of methylation-sensitive and insensitive isoschizomeric restriction enzyme pairs were selected, *i.e*., HpaII/MspI, AatII/FatI and BsmAI/BstCI (New England Biolabs, USA), and were used for digesting the pooled genomic DNA, representing each population, separately. Each restriction reaction was individually set up (final volume 25 μl) and contained 150 ng of the pooled genomic DNA, restriction enzyme (2 U), buffer (1 x), and sterile water (final volume upto 25 μl) and was incubated for 6 hrs at their optimum working temperatures (*i.e*., 37°C for HpaII, MspI and AatII; 55°C for BsmAI and FatI; 50°C for BstCI) to ensure complete digestion. Methylation-sensitive restriction enzymes HpaII, AatII and BsmAI were used to distinguish methylated DNA sequence from its unmethylated counterpart. Incubation was carried for 6 hrs at their respective optimum digestion temperatures to ensure complete digestion of unmethylated substrate sequences (*i.e*., 5’-CCGG-3’ for HpaII, 5’-GACGTC-3’ for AatII and 5’-GTCTC(1/5)-3’ for BsmAI) while keeping the methylated sequences intact. Based on this feature of the chosen restriction enzyme sets, comparative differential cleavage patterns for each of the selected genes between the BPH genomic DNAs from different populations were evaluated using semi-quantitative PCR.

### 2.5 Primer Design and PCR Amplification

To quantify methylation in different BPH populations, a semi-quantitative PCR assay was designed, which specifically targeted the predicted CpG islands of each of the five shortlisted BPH genes (*CYP6ER1, CYP6AY1, Endoglucanase, Carboxylesterase* and *Tf2-retrotransposon*). This assay was based on the premise that only genes possessing homogenously methylated alleles would remain unrestricted when methylation-sensitive restriction enzyme is used and, hence, can be amplified and quantified by PCR. In contrast, the partly methylated and unmethylated alleles would be digested/restricted by the restriction enzyme, resulting in the disruption of the target sequence and subsequent reduction in copy number, which can be quantified by semi-quantitative PCR.

**Fig. (1):**
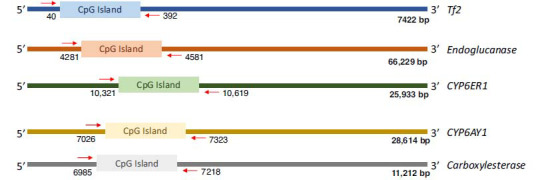
Diagrammatic representation of the gene length, location of CpG islands, and primer binding locations within the stress-responsive genes analyzed in the present study. Numbers represent nucleotide positions in the sequences.

In this regard, PCR primers for each gene selected in this study were designed from regions flanking the CpG island using the MacVector (Fig. **1**). The list of primer pairs used in this study is provided in Table **S1**. PCR amplification reactions were set up using the digested product as a template for all five genes to quantify and study DNA methylation. PCR reactions were carried out separately for each sample of digested DNAs (*i.e*., with methylation-sensitive and insensitive restriction enzymes), for each sample. While the amplification obtained from templates digested with the methylation-sensitive restriction enzyme allowed us to quantitate methylation levels across populations, the restriction reaction using methylation-insensitive restriction enzymes served as a reference to indicate the state of DNA methylation at the restriction site. Additionally, to normalise variations in the quantities of input template DNA within the PCR reactions and to determine the initial amounts of the target of interest, an independent reference (referred to as undigested control; UC) was also set up. All PCR reactions, with a final volume of 20 μl consisted of 200 μM dNTPs, 0.6 U Taq DNA polymerase (Bangalore Genei, (India) Pvt. Ltd.), 1X Taq buffer and 13 μM each of forward and reverse primers. For both the digested and undigested PCR, 15 ng of the genomic DNA was used as a template. The PCR amplification profile consisted of an initial denaturation at 95°C for 5 min, followed by 25 cycles (a reduced number of cycles ensured that the measurements of PCR products were made at the exponential phase) of denaturation at 95°C for 30 s, annealing at 55°C (for *CYP6AY1*, *Tf2*, *EG* and *Est*) or 60°C (for *CYP6ER1*) for 30 s, extension at 72°C for 30 s and a final extension of 72°C for 2 min. The PCR-amplified products (10 μl) were run on 1% agarose gel. A fixed amount of the PCR product representing each gene was loaded in each gel to normalise band intensities across gels.

### 2.6 Data Collection, Interpretation and Statistical Analysis

The quantity of PCR product obtained from both DNA templates (template restricted with methylation-sensitive and insensitive restrictions enzymes) after 25 cycles for each of the selected 5 genes was quantified, and the reactions were normalised based on the intensity of the PCR amplified fragment obtained for their respective undigested control (UC). The relative intensity for each fragment, in both methylation-sensitive and insensitive restriction lanes as well as undigested (control) lanes, was quantified using Image Lab software (v6.0.1; Bio-Rad Laboratories, USA). The band intensity was converted to nanograms of DNA by comparing it to the intensity of the fragments of a defined amount of the GeneRuler 1 kb Ladder (Thermo Fisher Scientific; Cat. no. SM0311) as standard. The variations in band intensities between methylation-sensitive lanes reflected the presence of heterogeneously methylated restriction sites. The band intensity or the quantity of amplified DNA obtained for methylation-sensitive reactions is directly proportional to the methylation status of the selected restriction site. Therefore, quantification of the bands in the sensitive lanes allowed estimation of the level of site-specific methylation across samples.

The intensity of the fragment resulting from PCR amplification of the undigested control (UC), which was not subjected to restriction digestion and thus exhibited no reduction in the copy number due to cleavage of the target site, represented 100% methylation. Based on this, the degree of methylation (defined as the fraction of methylated alleles) for each sample was determined by dividing the band intensity values (ng DNA) obtained for each sample by the intensity value for its corresponding UC (reference). Further, the methylation data obtained for each gene were subjected to statistical analyses to assess the significance of the observed variations in percent methylation (vis-à-vis the five targeted loci) between and within BPH populations.

One-way ANOVA analysis was carried out using SPSS Statistics (version 22.0.) to identify genes that varied significantly with regard to 5 mC methylation across the six BPH populations analysed. Genes that showed significant variation in their methylation status across seasons were identified from the ones that varied between years. Further, the data were subjected to multivariate analysis to identify genes exhibiting significant variability across both independent variables, *i.e*., between seasons and years. Here, the impact of independent variables (predictors), *i.e*., months and years, on the methylation status (dependent/response variable) was analysed. The results were validated by Fisher's least significant difference (LSD) post hoc tests to uncover specific differences between populations and identify the variables differentiating these populations. The percent epigenetic polymorphism across BPH populations was estimated by k-means clustering analysis. In addition, the same software was used to perform Spearman’s correlation analysis (2- tailed test, *p*-value cut-off < 0.05) for assessing the strength of dependence or influence of genes on one another vis-à-vis their methylation status.

## 3 RESULTS

**Fig. (2):**
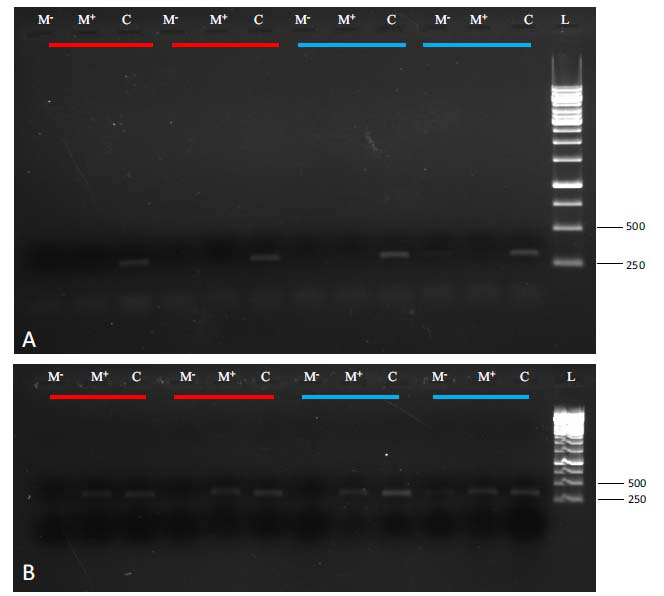
Agarose gel depicting variations in amplifications of a portion of *CYP6AY1* PCR amplified from different BPH individuals representing populations collected during different seasons and years (refer to Materials and Methods for details). The PCR amplifications were carried out using BPH genomic DNA templates restricted with a methylation-sensitive (M^+^) or insensitive (M^-^) restriction enzyme. Unrestricted DNA (C) served as a control. Cyan and orange bars (above lanes) represent the BPH population collected in 2017 and 2018, respectively. Duplicates for each population are represented by same-colored bars. Panel (**A**) shows methylation status (hypomethylated state; absence of bands in both M+ and M- lanes indicate that the CpG site is unmethylated and hence is digested by both the restriction enzymes) of BPH samples collected in June, and panel (**B**) shows the methylation status (hypermethylated state, presence of a band in M+ indicates that the site is methylated and hence is protected from restriction digestion) of BPH samples collected during August. L: 1-kb ladder as a molecular mass marker. The figures on the right represent molecular masses in base pairs (bp).

**Fig. (3):**
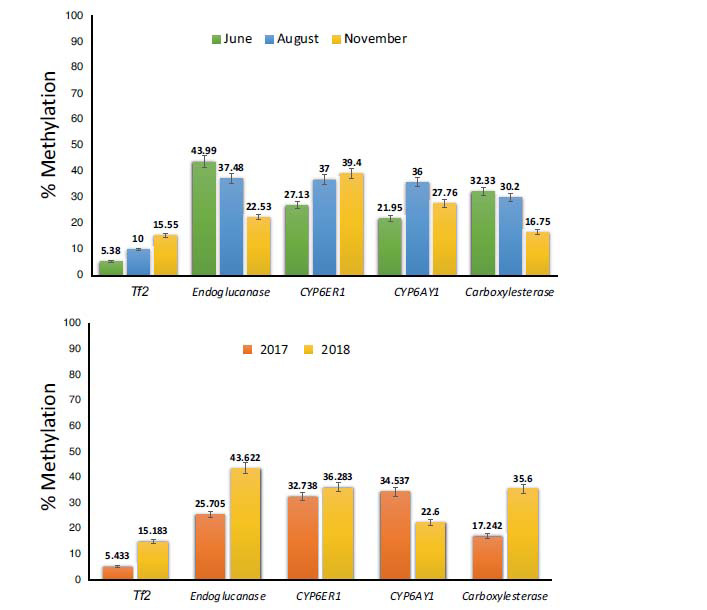
Bar plots showing the mean percent methylation of the selected stress-responsive genes in the BPH populations (**A**) across different months. (**B**) across years. Error bars represent mean ± SD. The analysis included two replicates for each population (see ‘Materials and Methods’ section for details).

The CpG islands were detected mainly within the gene body of the stress-responsive genes analysed in the present study. They contained several regulatory motifs and transcription factor binding sites (for details also see Fig. **3** in Gupta and Nair, 2022 [9]). Using methylation-sensitive restriction enzymes combined with semi-quantitative PCR allowed rapid estimation of the degree of DNA methylation (of selected stress-responsive genes) between and within BPH populations without treating the DNA samples with sodium bisulphite. Methylation-sensitive amplification profiles, generated using a combination pair of methylation-sensitive/insensitive restriction enzymes, revealed high levels of epigenetic diversity between BPH populations with regard to the genes analysed in the present study (Figs. **2** and **3**). Besides differences among populations, variation within populations could also be detected (Fig. **2** and Table **S2**). Differences in the intensity of bands across methylation-sensitive restriction reactions indicated the presence of different states of cytosine methylation at the restriction sites across BPH populations (Fig. **2**). In addition, we observed variations for the insensitive lanes (*i.e*., restriction with methylation insensitive restriction enzyme), which indicated the existence of genetic variation at the specific locus leading to the loss of restriction enzyme recognition site and/or inhibition of DNA restriction at the specific restriction sites surveyed in the present study (Fig. **S1**).

Results revealed that BPH genes exhibit low levels of methylation under field conditions, as the average percent methylation estimated across all BPH populations for *Tf2, Endoglucanase, CYP6ER1, CYP6AY1* and *Carboxylesterase* was 10.308, 34.663, 34.511, 28.568, and 26.424, respectively (Fig. **S2**). Further, the percent epigenetic polymorphism across populations (as determined by comparing the restriction amplification profile obtained using a specific methylation-sensitive enzyme across populations) varied between loci (Figs. **3** and **S3**). Significant differences in the methylation status were observed across months and years (Fig. **3**). Cluster analysis using K-means clustering revealed discrete cluster centroids for the methylation values observed for *Tf2* (6.1% and 13.3%), *Endoglucanase* (21.5% and 44.1%), *Carboxylesterase* (14.3% and 35.1%), *CYP6ER1* (33.8% and 35.5%) and *CYP6AY1 (*21.86% and 37.96%) across BPH populations (Table **S3** and Fig. **S4**).

Additionally, results obtained using one-way ANOVA, performed to compare the effect of ‘year’ and ‘month’ of collection on the methylation status of genes, indicated that while the methylation status of *Carboxylesterase* varied significantly between BPH populations sampled in the year 2017 and 2018 (F value 5.959; *p* ≤ 0.05), *CYP6ER1* showed significant seasonal variation, *i.e*., across months (F value 5.168; *p* ≤ 0.05) (Table **S2**). However, in the case of *CYP6AY1*, *Endoglucanase,* and *Tf2,* the variation within groups was higher than that observed between groups and, hence, was deemed non-significant (Table **S2**).

To identify, interpret and model the linear relationship between and among experimental variables (including both independent variables, ‘Years’ and ‘Months’), multivariate analysis was carried out, and results revealed that while the methylation status of *Tf2* (F value 44.338; *p* ≤ 0.05) and *CYP6AY1* (F value 4.901; *p* ≤ 0.05) was significantly influenced by both ‘month’ and ‘year’ of collection, *Endoglucanase* (F value 6.925; *p* ≤ 0.05) and *Carboxylesterase* (F value 6.866; *p* ≤ 0.05) typically exhibited yearly variation and *CYP6ER1* (F value 6.624; *p* ≤ 0.05) varied across months. Further, it was found that the ‘month’ of the collection had a higher contribution to the overall epigenetic variation observed between BPH populations (Pillai’s Trace value significant at a 5% level as the observed *p*-value is 0.01) as compared to the ‘year’ (Pillai’s Trace value non-significant at a 5% level as the observed *p*-value is 0.125). Furthermore, the variance in the eta squared values of Pillai’s Trace for *Tf2, CYP6AY1, CYP6ER1, Endoglucanase,* and *Carboxylesterase* were 0.56, 0.24, 0.103, 0.058 and 0.029, respectively, indicating that *Tf2* accounted for the maximum (56%) and *Carboxylesterase* (2.9%) accounted for the minimum epigenetic variability observed between BPH populations (Table **2**). These results were further validated by post hoc tests, which confirmed the presence of significant variation in the methylation status of genes analysed in the present study across BPH populations sampled during different months (Table **S4**). Besides, the analysis also indicated that while the methylation of *Tf2* varied across all three months (*i.e*., between June, August and November), *Endoglucanase, CYP6ER1*, and *CYP6AY1* showed a gradual shift in their methylation status from June to November. However, as previously indicated by the multivariate tests (stated above), *Carboxylesterase* did not exhibit significant change and hence had the least contribution to the observed epigenetic variance between BPH populations among the genes tested (Table **2** and **S4**).

**Fig. (4):**
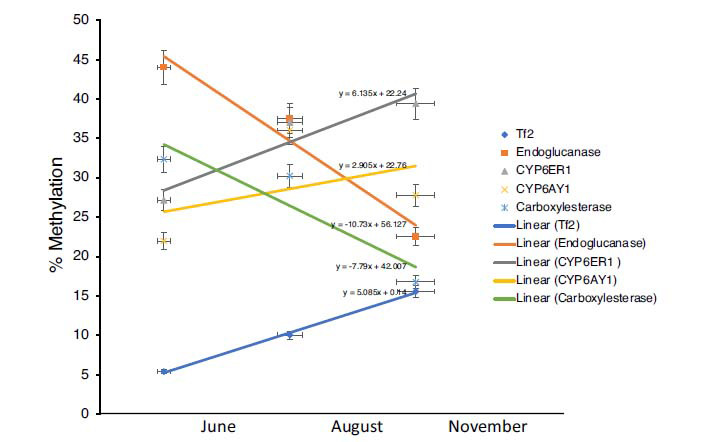
Trend plot illustrating seasonal changes in the methylation status of stress-responsive genes across BPH populations collected during different periods. The horizontal axis represents the months in which the BPH samples were sampled, and the vertical axis indicates percent methylation. For 3 of the 5 genes studied, an upward trend for DNA methylation was observed from June to November.

**Fig. (5):**
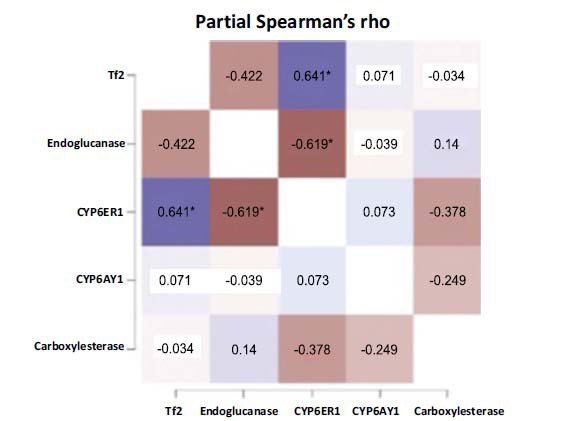
Correlation heat map representation of a pairwise correlation analysis indicating inter-relatedness of selected genes of field-collected BPH populations with regard to their methylation status. Blue represents a positive correlation, while brown signifies a negative correlation between variables (genes).

Further, it was observed that while the methylation status of *CYP6ER1*, *CYP6AY1* and *Tf2* significantly increased across months (*i.e*., from June to November), it showed a gradual dip for *Endoglucanase* and *Carboxylesterase* (Fig. **4**). Additionally, to explore the relatedness of genes with regard to the observed epigenetic diversity in the field populations of BPH, we performed pairwise-correlation estimations and results indicated that the methylation status of *Tf2* exhibited a positive correlation with that of *CYP6ER1* (Spearman’s rho value 0.641; *p*-value 0.034) which in turn showed negative correlation with *Endoglucanase* (Spearman’s rho value -0.619; *p*-value 0.042) (Fig. **5** and Table **S5**).

## 4 DISCUSSION

This study focussed on estimating the relative site-specific DNA methylation for five stress-responsive genes in BPH populations across different rice growing seasons and years. PCR amplification profiles (obtained for the methylation-sensitive restriction reactions) for *CYP6AY1, CYP6ER1, Carboxylesterase, Tf2*, and *Endoglucanase* revealed significant epigenetic diversity in the field-collected BPH populations (Figs. **2** and **3**). Variations were also observed in the PCR amplification profiles for the methylation-insensitive reactions (Fig. **S1**), likely due to genetic diversity (allelic variations) at some restriction sites and/or non-CG methylation in the BPH genome (Fig. **S5**). Our earlier studies have shown that BPH possesses methylation in all three contexts (*i.e*., CG, CHG and CHH [9, 31]. Therefore, these results are highly likely to reflect changes in methylation patterns in non-CG contexts across BPH populations. Indications of such a possibility and also accounting for the presence of a band in the insensitive (M^-^) lane is the fact that while the methylation insensitive restriction enzyme (MspI) used in the present study can efficiently cleave non-methylated 5’ CCGG 3’ sequences and hemi- (*i.e*., mC in one DNA strand only) or fully-methylated 5’ CmCGG 3’ sequences (*i.e*., methylation of inner cytosine), it cannot cleave the hemi- and fully-methylated mCCGG and mCmCGG sequences [42].

Further, our data showed that BPH exhibits a low methylation level under field conditions (Fig. **S2**, [9];). However, even at low levels, DNA methylation can significantly regulate BPH’s fitness in changing environments. Gene body methylation in insects is known to modulate gene expression, exon shuffling and alternative splicing [43, 44]. In our study, four of the five analysed restriction sites are located in the exonic region of the corresponding genes (see Table **S6**), indicating that modification(s) in DNA methylation at these sites can influence BPH’s phenotype. This is corroborated by our recent study, where we show how methylation/demethylation at these sites affects gene activity and contributes to BPH’s phenotypic plasticity under changing environmental conditions [9].

Next, the percent of epigenetic polymorphism varied between loci (Fig. **S3** and Table **S7**), suggesting that methylation and demethylation of genes are influenced by their functional relevance and importance for BPH’s survival. Further, even though discrete cluster centroids were obtained from the k-means clustering analysis of the methylation values of *CYP6AY1, CYP6ER1, Carboxylesterase, Tf2,* and *Endoglucanase* across BPH populations, the distance between the cluster centres varied between genes, indicating that each gene possessed different levels of epigenetic diversity (Table **S3** and Fig. **S4**). These variations can be exploited to distinguish morphologically indistinguishable BPH individuals by identifying additional methylation-based epiallelic markers from other regions of the BPH genome. The identification of such markers holds implications for the integrated management of BPH.

Furthermore, we studied the impact of ‘seasons’ on DNA methylation patterns and its contribution to seasonal polymorphisms in BPH populations [8, 10-15, 21]. Sampling was conducted in different months (June, August, and November) during the rice-growing period for two consecutive years (2017 and 2018). To observe the effect of each variable (*i.e*., ‘year’ and ‘month’) on the methylation status of genes, the data were subjected to one-way ANOVA analysis. Results indicated that while the methylation status of *Carboxylesterase* differed significantly between BPH populations sampled in the years (2017 and 2018), *CYP6ER1* showed significant seasonal variation, *i.e*., across months (Table **S2**). However, *CYP6AY1*, *Endoglucanase,* and *Tf2* displayed high intra-population variation and were deemed non-significant. Additionally, to evaluate the combined influence of year and month on the methylation of *CYP6AY1*, *Endoglucanase,* and *Tf2*, we resorted to multivariate tests.

The results of the multivariate analysis revealed that both ‘month’ and ‘year’ significantly influenced the methylation status of *Tf2* and *CYP6AY1,* while *Endoglucanase* and *Carboxylesterase* exhibited yearly variation and *CYP6ER1* varied across months. Among the studied genes, *Tf2* displayed the highest (56%) and *Carboxylesterase* (2.9%), the lowest epigenetic variability between BPH populations (Table **2**). Here, it is worth noting that *Tf2,* being an LTR-retrotransposon with multiple insertions in the BPH genome [31], is likely to exhibit high polymorphism due to varying methylation patterns in different insertions. Besides, as the primers used to carry out the PCR assay were not specific to a particular *Tf2* insertion in the BPH genome, therefore, amplification of this locus was not restricted to one or a few insertional copies but to all the elements that contain the primer binding sites, thereby accounting for the high variation observed for *Tf2*. Further, the pairwise correlation estimations for all five genes indicated a positive correlation between the methylation status of Tf2 and *CYP6ER1*. *Tf2* being a transposable element, can spread the epigenetic marks to neighbouring genes [45, 46], and as ~50% of *Tf2* insertions in the BPH genome are flanked by *CYP6ER1*, [31] this correlation could be attributed to their genomic proximity. However, further investigation is required to confirm and establish if and how the methylation status of these loci is correlated.

Interestingly, while *CYP6AY1* showed variation between ‘years’ and ‘months’, *CYP6ER1* exhibited seasonal changes. Both genes belong to the family of P450 monooxygenases and are largely involved in pesticide detoxification in BPH [47]. *CYP6AY1* is effective in metabolizing various pesticides, while *CYP6ER1* is particularly responsive to imidacloprid (a neonicotinoid pesticide commonly used against BPH). Earlier studies have shown high expression levels of *CYP6ER1* in pesticide-resistant BPH populations [47, 48]. Therefore, and as reported in our earlier study [9], the differences in the methylation levels observed in BPH populations across the rice-growing season can contribute to variations in the activity of *CYP6AY1* and *CYP6ER1*, potentially affecting their pesticide resistance/tolerance capacities. Hence, results from this study can also be utilised to develop a rapid evaluation method for assessing the pesticide resistance/tolerance capacity of a BPH population infesting a rice field by estimating their methylation status using the methods described here.

Further, *EG* and *Est* exhibited minimum variation in their methylation status (over ‘months’ and ‘years’) and showed the least epigenetic variance between populations (Table **2**). It is worth noting that BPH constitutively requires both *EG* and *Est* for its survival. While the former assists BPH in overcoming plant cell wall defence metabolites, enabling it to feed on rice plants, the latter plays an important role in xenobiotic detoxification. Hence, these are regarded as vital and indispensable genes for BPH survival [49, 50], and therefore, it is highly likely that relatively uniform levels of methylation are maintained for *EG* and *Est* across BPH populations. However, the minimal variation observed for these genes could either be an outcome of inherent genetic differences between populations or differences in their nutritional intake (host plant or rice varieties) and/or other environmental exposures, which warrant further investigation.

A comparison of the effect of ‘month’ and ‘year’ on the overall epigenetic variation revealed that the ‘month’ of the collection had a higher impact on the observed epigenetic diversity between BPH populations, suggesting the involvement of epigenetic changes in mediating seasonal polymorphisms in BPH. Post hoc tests confirmed the reliability and significance of these results (see Table **S4**), validating the significant variation in the methylation status of genes analysed across BPH populations sampled during different months. Further, the analysis showed that while the methylation of *Tf2* varied across all three months (*i.e*., between June, August, and November), *EG, CYP6ER1,* and *CYP6AY1* exhibited a gradual shift in their methylation status from June to November. As previously indicated by the multivariate analyses, *Est* did not exhibit a significant change in its methylation status and contributed least to the epigenetic variance between BPH populations (Tables **2** and **S4**).

The analysis of the patterns of methylation changes at the restriction sites analysed in the present study showed that the methylation value of cytosine increased significantly from June to November for *CYP6ER1*, *CYP6AY1* and *Tf2* but decreased for *EG* and *Carboxylesterase* (Fig. **4**). This implied that BPH populations with different epigenetic profiles are found in Delhi across seasons. These differences can be attributed to the continuous inflow of different BPH populations through migration from distant regions throughout the rice-growing season. However, it is equally probable that the observed fluctuations in the epigenetic status of BPH populations collected during different months of the year, are a survival strategy deployed by the BPH to adapt to changing climatic conditions across seasons. In the Northern regions of India, rice is primarily sown during May-June and harvested during October-November, implying that rice is not available for the monophagous BPH to feed on throughout the year. Therefore, variations in the methylation status of feeding-related genes (*e.g*., *EG*) are likely linked to the feeding patterns of BPH. Likewise, pesticide application in paddy fields varies during the different stages of rice cultivation. Therefore, we speculate that the methylation status of pesticide detoxification genes (such as *CYP6AY1*, *CYP6ER1,* and *Est*) in a BPH population is related to its extent of exposure to pesticides, which varies across seasons. Collectively, these observations imply that the observed variations in DNA methylation in BPH populations are probably induced in response to external environmental stimuli.

The assay employed in this study allowed rapid estimation of the DNA methylation at the targeted CpG sites without requiring sodium bisulfite treatment of the DNA samples, which is tedious, time-consuming and expensive. This method relied on the differential sensitivity of isoschizomeric restriction enzymes and subsequent PCR amplification to detect site-specific cytosine methylation status within the CpG island. However, a major limitation of MSAP-based approaches is their inability to quantify DNA methylation accurately, mainly when DNA methylation exhibits cell-to-cell heterogeneity (*i.e*., differential epigenetic status of the genome among cells) [51, 52], causing genes in a particular sample to display varying degrees of methylation. In such cases, bulk analyses may be less sensitive in determining the exact methylation status of the assayed locus. However, as our primary objective was to determine whether BPH populations found across the rice-growing season are epigenetically diverse; therefore, methylation-sensitive restriction enzyme analysis (MSRA) coupled with semi-quantitative PCR proved to be a useful, reliable, cost-effective and quick method to estimate the extent of DNA methylation of the target locus. Besides being cost-effective, this technique could be easily applied to study the methylation state of individual insects in a population to obtain statistically significant and reproducible data. Several pestilent outbreaks of insect pests of agricultural importance (such as BPH) can be prevented if we have prior knowledge of its likely occurrence. Therefore, the identification of methylation-based epiallelic markers in the wild populations of BPH has implications for the assessment of prevailing epigenetic variations and adaptive capabilities. Moreover, this information can be subsequently used to devise appropriate pest management strategy(ies) for field use.

## CONCLUSION

By comparing epigenetic variations in five genes in the field populations of BPH, this study, for the first time, revealed useful information regarding the epigenetic component of variations prevalent in BPH. We explored epigenetic diversity, structure and differentiation in field populations of BPH collected across the rice-growing seasons over two consecutive years. Though our findings suggest that epigenetic (methylation/demethylation) mechanisms might play an important role in how BPH regulates its genes in time and space, extending this study to individual insects collected from different months and years could give us a better estimate of the level of seasonal epigenetic diversity prevalent in BPH populations. Besides, the finer intricacies of how these molecular processes work in BPH is an aspect that requires further investigation. In addition, data obtained also indicated that morphologically indistinguishable populations of BPH could be epigenetically distinct. Therefore, field screenings of BPH populations to assess prevailing epigenetic variations not only added valuable insights into the possibility of epigenetic differentiation of BPH populations but can also help unravel the importance and role of DNA methylation in explaining phenotypic variation(s). Undoubtedly, while it is important to delineate the levels of epigenetic diversity in BPH populations, it would be equally important to ascertain whether the degree of epigenetic variation observed across BPH populations can substantially contribute to the phenotypic variation and eventually lead to better adaptability of these populations to varied environments and host plant varieties. While we studied DNA methylation to assess epigenetic diversity, it is important to note that other epigenetic mechanisms interact with DNA methylation, and altogether, this interactive and dynamic epigenetic machinery can enhance diversity. In this regard, the findings from this study can form the basis for extending the investigation to other regions of the BPH genome and across individuals collected from different geographical locations and life stages to understand better the role(s) of DNA methylation and other epigenetic processes in conferring phenotypic plasticity to BPH.

## AUTHORS’ CONTRIBUTIONS

Ayushi Gupta contributed to the investigation, formal analysis, data curation, visualization, and writing of the original Draft. Suresh Nair assisted in conceptualization, investigation, supervision, writing, review and editing, and funding acquisition.

## Figures and Tables

**Table 1:** List of stress-responsive genes selected for the present study for estimating their relative site-specific DNA methylation (5 mC) levels across BPH populations and their functional relevance in BPH.

**Genes**	**Functional Relevance for BPH**	**References**
*CYP6ER1*	P450 monooxygenase involved in imidacloprid (pesticide) metabolism	Bao *et al.*, 2016 [47]; Pang *et al.*, 2016 [48]
*CYP6AY1*	P450 monooxygenase involved in pesticide degradation	Bao *et al.*, 2016 [47]
*Endoglucanase*	Plant cell wall degrading enzyme; facilitates BPH feeding on rice host	Ji *et al.*, 2017 [49]
*Carboxylesterase*	Involved in xenobiotic detoxification	Mao *et al.*, 2021 [50]
*Tf2*	LTR retrotransposon flanking stress-responsive genes in the BPH genome	Gupta and Nair, 2021 [31]

**Table 2(a):** Results of the multivariate analysis in site-specific methylation of selected genes across BPH populations, along with month and year of collection.

**Effect**	**Dependent Variable**	**F statistics**	***p*-value**	**Partial Eta Squared**	**Observed Power**
Year	*Tf2*	62.828	0.000*	0.913	1.000
	*Endoglucanase*	6.952	0.039*	0.537	0.598
	*CYP6ER1*	1.478	0.270	0.198	0.177
	*CYP6AY1*	13.162	0.011*	0.687	0.854
	*Carboxylesterase*	6.866	0.040*	0.534	0.592
Month	*Tf2*	22.871	0.002*	0.884	0.997
	*Endoglucanase*	3.497	0.098	0.538	0.434
	*CYP6ER1*	6.624	0.030*	0.688	0.705
	*CYP6AY1*	6.143	0.035*	0.672	0.671
	*Carboxylesterase*	1.937	0.224	0.392	0.261
Month * Year	*Tf2*	44.338	0.000*	0.937	1.000
	*Endoglucanase*	1.301	0.339	0.302	0.188
	*CYP6ER1*	2.029	0.212	0.403	0.272
	*CYP6AY1*	4.901	0.055	0.620	0.571
	*Carboxylesterase*	0.824	0.483	0.216	0.135

**Note:** *The mean difference is significant at the *p* < 0.05

**Table 2(b):** Estimation of Eta square values using the results obtained from the multivariate analysis.

**Genes**	**Partial Eta _Year**	**Eta** **Year**	**Variance**	**Partial Eta_** **Month**	**Eta** **Months**	**Variance**	Year*Month_	**Eta square**	**Variance**
***Tf2***	0.913	0.833	0.434	0.884	0.781	0.363	0.937	0.877	0.561
***Endoglucanase***	0.537	0.288	0.150	0.538	0.289	0.134	0.302	0.091	0.058
***CYP6ER1***	0.198	0.039	0.020	0.688	0.473	0.220	0.403	0.162	0.103
***CYP6AY1***	0.687	0.471	0.246	0.672	0.451	0.210	0.62	0.384	0.245
***Carboxylesterase***	0.534	0.285	0.148	0.392	0.153	0.071	0.216	0.046	0.029

## Data Availability

All data needed to evaluate the conclusions in the paper are presented in the paper and/or the Supplementary Materials. The raw data files for the image analysis obtained from the quantification software are available on Zenodo under the doi 10.5281/zenodo.7963141.

## LIST OF ABBREVIATIONS

BPH: Brown Planthopper
DNMT: DNA Methyltransferase
LSD: Least Significant Difference
MSRA: Methylation-sensitive Restriction Assay
UC: Undigested Control

## References

[1] Liu Y., Chen L., Liu Y., Dai H., He J., Kang H., Pan G., Huang J., Qiu Z., Wang Q., Hu J., Liu L., Chen Y., Cheng X., Jiang L., Wan J. (2016). Marker assisted pyramiding of two brown planthopper resistance genes, *Bph3* and *Bph27* (*t*), into elite rice cultivars.. Rice.

[2] Bentur J.S., Sain M., Kalode M.B. (1982). Studies on egg and nymphal parasites of rice planthoppers, *Nilaparvata lugens* (Stål) and *Sogatella furcifera* (Horvath). *Proc. Indian Acad. Sci.*. Anim. Sci..

[3] Aktar W., Sengupta D., Chowdhury A. (2009). Impact of pesticides use in agriculture: their benefits and hazards.. Interdiscip. Toxicol..

[4] Hawkins N.J., Bass C., Dixon A., Neve P. (2019). The evolutionary origins of pesticide resistance.. Biol. Rev. Camb. Philos. Soc..

[5] Zhang X., Liu X., Zhu F., Li J., You H., Lu P. (2014). Field evolution of insecticide resistance in the brown planthopper (*Nilaparvata lugens* Stål) in China.. Crop Prot..

[6] Rosenberg L.J., Magor J.I. (1987). Predicting windborne displacements of the brown planthopper, *Nilaparvata lugens* from synoptic weather data: Long-distance displacements in the North-East monsoon.. J. Anim. Ecol..

[7] Barnes L. (2021). Insect developmental plasticity: The role in a changing environment.. Cardinal Edge.

[8] Pender J. (2009). Migration of the brown planthopper, *Nilaparvata lugens* (Stal.) with special reference to synoptic meteorology.. Grana.

[9] Gupta A., Nair S. (2022). Heritable epigenomic modifications influence stress resilience and rapid adaptations in the brown planthopper (*Nilaparvata lugens*).. Int. J. Mol. Sci..

[10] Satpathi C.R., Katti G., Prasad Y.G., Krishi Viswavidyalaya C., Mohanpur P.O. (2011). Effect of seasonal variation on life table of brown planthopper *Nilaparvata lugens* Stål on rice plant in Eastern India.. Middle East J. Sci. Res..

[11] Claridge M.F., Hollander J.D., Morgan J.C. (1985). The status of weed-associated populations of the brown planthopper, *Nilaparvata lugens* (Stål) - host race or biological species?. Zool. J. Linn. Soc..

[12] Nagata T., Masuda T. (1980). Insecticide susceptibility and wing-form ratio of the brown planthopper, *Nilaparvata lugens* (Stål) (Hemiptera: Delphacidae) and the white backed planthopper, *Sogatella furcifera* (Horvath) (Hemiptera: Delphacidae) of Southeast Asia.. Appl. Entomol. Zool..

[13] Matsumura M., Takeuchi H., Satoh M., Sanada-Morimura S., Otuka A., Watanabe T., Van Thanh D. (2008). Species-specific insecticide resistance to imidacloprid and fipronil in the rice planthoppers *Nilaparvata lugens* and *Sogatella furcifera* in East and South-east Asia.. Pest Manag. Sci..

[14] Wada T., Ito K., Takahashi A., Tang J. (2007). Variation of pre-ovipositional period in the brown planthopper, *Nilaparvata lugens*, collected in tropical, subtropical and temperate Asia.. J. Appl. Entomol..

[15] Matsumoto Y., Matsumura M., Sanada-Morimura S., Hirai Y., Sato Y., Noda H. (2013). Mitochondrial *cox* sequences of *Nilaparvata lugens* and *Sogatella furcifera* (Hemiptera, Delphacidae): low specificity among Asian planthopper populations.. Bull. Entomol. Res..

[16] Moczek A.P. (2010). Phenotypic plasticity and diversity in insects.. Philos. Trans. R. Soc. Lond. B Biol. Sci..

[17] Fusco G., Minelli A. (2010). Phenotypic plasticity in development and evolution: facts and concepts.. Philos. Trans. R. Soc. Lond. B Biol. Sci..

[18] Whitman D.W., Agrawal A.A., Whitman D.W. (2009). What is phenotypic plasticity and why is it important?. Phenotypic Plasticity of Insects..

[19] Hochkirch A., Deppermann J., Gröning J. (2008). Phenotypic plasticity in insects: The effects of substrate color on the coloration of two ground-hopper species.. Evol. Dev..

[20] Srinivasa N., Chander S., Twinkle, Chandel R.K. (2020). Genetic homogeneity in brown planthopper, *Nilaparvata lugens* (Stål) as revealed from mitochondrial cytochrome oxidase I.. Curr. Sci..

[21] Gupta A., Sinha D.K., Nair S. (2022). Shifts in *Pseudomonas* species diversity influence adaptation of brown planthopper to changing climates and geographical locations.. iScience.

[22] Glastad K.M., Hunt B.G., Goodisman M.A.D. (2019). Epigenetics in insects: Genome regulation and the generation of phenotypic diversity.. Annu. Rev. Entomol..

[23] Lo N., Simpson S.J., Sword G.A. (2018). Epigenetics and developmental plasticity in orthopteroid insects.. Curr. Opin. Insect Sci..

[24] Jing S., Zhang L., Ma Y., Liu B., Zhao Y., Yu H., Zhou X., Qin R., Zhu L., He G. (2014). Genome-wide mapping of virulence in brown planthopper identifies loci that break down host plant resistance.. PLoS One.

[25] Moore L.D., Le T., Fan G. (2013). DNA methylation and its basic function.. Neuropsychopharmacology.

[26] Provataris P., Meusemann K., Niehuis O., Grath S., Misof B. (2018). Signatures of DNA methylation across insects suggest reduced DNA methylation levels in Holometabola.. Genome Biol. Evol..

[27] Bewick A.J., Vogel K.J., Moore A.J., Schmitz R.J. (2017). Evolution of DNA methylation across insects.. Mol. Biol. Evol..

[28] Zhang J., Xing Y., Li Y., Yin C., Ge C., Li F. (2015). DNA methyltransferases have an essential role in female fecundity in brown planthopper, *Nilaparvata lugens.*. Biochem. Biophys. Res. Commun..

[29] Nguyen N.D., Matsuura T., Kato Y., Watanabe H. (2021). DNMT3.1 controls trade-offs between growth, reproduction, and life span under starved conditions in *Daphnia magna.*. Sci. Rep..

[30] Loughland I., Little A., Seebacher F. (2021). DNA methyltransferase 3a mediates developmental thermal plasticity.. BMC Biol..

[31] Gupta A., Nair S. (2021). Methylation patterns of Tf2 retrotransposons linked to rapid adaptive stress response in the brown planthopper (*Nilaparvata lugens*).. Genomics.

[32] Cao J.X., Zhang H.P., Du L.X. (2013). Influence of environmental factors on DNA methylation.. Yi Chuan.

[33] Pegoraro M., Bafna A., Davies N.J., Shuker D.M., Tauber E. (2016). DNA methylation changes induced by long and short photoperiods in *Nasonia*.. Genome Res..

[34] Lafuente E., Beldade P. (2019). Genomics of developmental plasticity in animals.. Front. Genet..

[35] Ashe A., Colot V., Oldroyd B.P. (2021). How does epigenetics influence the course of evolution?. Philos. Trans. R. Soc. Lond. B Biol. Sci..

[36] Singer-Sam J., Grant M., LeBon J.M., Okuyama K., Chapman V., Monk M., Riggs A.D. (1990). Use of a HpaII-polymerase chain reaction assay to study DNA methylation in the Pgk-1 CpG island of mouse embryos at the time of X-chromosome inactivation.. Mol. Cell. Biol..

[37] Herman J.G., Graff J.R., Myöhänen S., Nelkin B.D., Baylin S.B. (1996). Methylation-specific PCR: A novel PCR assay for methylation status of CpG islands.. Proc. Natl. Acad. Sci. USA.

[38] Oakes C.C., La Salle S., Robaire B., Trasler J.M. (2006). Evaluation of a quantitative DNA methylation analysis technique using methylation-sensitive/dependent restriction enzymes and real-time PCR.. Epigenetics.

[39] Beikircher G., Pulverer W., Hofner M., Noehammer C., Weinhaeusel A. (2018). Multiplexed and sensitive DNA methylation testing using methylation-sensitive restriction enzymes “MSRE-qPCR”.. Methods Mol. Biol..

[40] Perry N., Wasko K., Cheng J., Tabbaa D., Marco E., Giannoukos G., Albright C.F., Borges C.M. (2021). Methylation-sensitive restriction enzyme quantitative polymerase chain reaction enables rapid, accurate, and precise detection of methylation status of the regulatory t cell (treg)-specific demethylation region in primary human tregs.. J. Immunol..

[41] Maniatis T., Fritsch E.F., Sambrook J., Wood E.J. (1982). Molecular cloning. A laboratory manual.. Biochemical education..

[42] Fulneček J., Kovařík A. (2014). How to interpret methylation sensitive amplified polymorphism (MSAP) profiles?. BMC Genet..

[43] Bonasio R., Li Q., Lian J., Mutti N.S., Jin L., Zhao H., Zhang P., Wen P., Xiang H., Ding Y., Jin Z., Shen S.S., Wang Z., Wang W., Wang J., Berger S.L., Liebig J., Zhang G., Reinberg D. (2012). Genome-wide and caste-specific DNA methylomes of the ants. *Camponotus floridanus* and *Harpegnathos saltator.*. Curr. Biol..

[44] Xu G., Lyu H., Yi Y., Peng Y., Feng Q., Song Q., Gong C., Peng X., Palli S.R., Zheng S. (2021). Intragenic DNA methylation regulates insect gene expression and reproduction through the MBD/Tip60 complex.. iScience.

[45] Choi J.Y., Lee Y.C.G. (2020). Double-edged sword: The evolutionary consequences of the epigenetic silencing of transposable elements.. PLoS Genet..

[46] Chown S.L., Terblanche J.S. (2006). Physiological diversity in insects: Ecological and evolutionary contexts.. Adv. Insect Physiol..

[47] Bao H., Gao H., Zhang Y., Fan D., Fang J., Liu Z. (2016). The roles of *CYP6AY1* and *CYP6ER1* in imidacloprid resistance in the brown planthopper: Expression levels and detoxification efficiency.. Pestic. Biochem. Physiol..

[48] Pang R., Chen M., Liang Z., Yue X., Ge H., Zhang W. (2016). Functional analysis of *CYP6ER1*, a P450 gene associated with imidacloprid resistance in *Nilaparvata lugens.*. Sci. Rep..

[49] Ji R., Ye W., Chen H., Zeng J., Li H., Yu H., Li J., Lou Y. (2017). A salivary endo-β-1,4-glucanase acts as an effector that enables the brown planthopper to feed on rice.. Plant Physiol..

[50] Mao K., Ren Z., Li W., Cai T., Qin X., Wan H., Jin B.R., He S., Li J. (2021). Carboxylesterase genes in nitenpyram-resistant brown planthoppers, *Nilaparvata lugens*.. Insect Sci..

[51] Smallwood S.A., Lee H.J., Angermueller C., Krueger F., Saadeh H., Peat J., Andrews S.R., Stegle O., Reik W., Kelsey G. (2014). Single-cell genome-wide bisulfite sequencing for assessing epigenetic heterogeneity.. Nat. Methods.

[52] Huan Q., Zhang Y., Wu S., Qian W. (2018). HeteroMeth: A database of cell-to-cell heterogeneity in DNA methylation.. Genomics Proteomics Bioinformatics.

