# Supplementary material for: Epigenetic Diversity Underlying Seasonal and Annual Variations in Brown Planthopper (BPH) Populations as Revealed by Methylation- sensitive Restriction Assay
Source: Curr Genomics. 2023 Dec 28;24(6):354–67. doi: 10.2174/0113892029276542231205065843 (PMC10845068; doi:10.2174/0113892029276542231205065843)
Supplement: Supplementary file 1 [file CG-24-354_SD1.zip › CG-24-354/3b-BMS-CG-2023-132 Supplementary Material.docx]

Supplementary Material

Epigenetic Diversity Underlying Seasonal and Annual Variations in Brown Planthopper (BPH) Populations as Revealed by Methylation-sensitive Restriction Assay

Ayushi Gupta^1,2^ and Suresh Nair^1,*^

^1^Plant-Insect Interaction Group, International Centre for Genetic Engineering and Biotechnology (ICGEB), Aruna Asaf Ali Marg, New Delhi, 110067, India; ^2^Current Address: Institute of Molecular Plant Sciences, University of Edinburgh, Edinburgh EH 93BF, UK


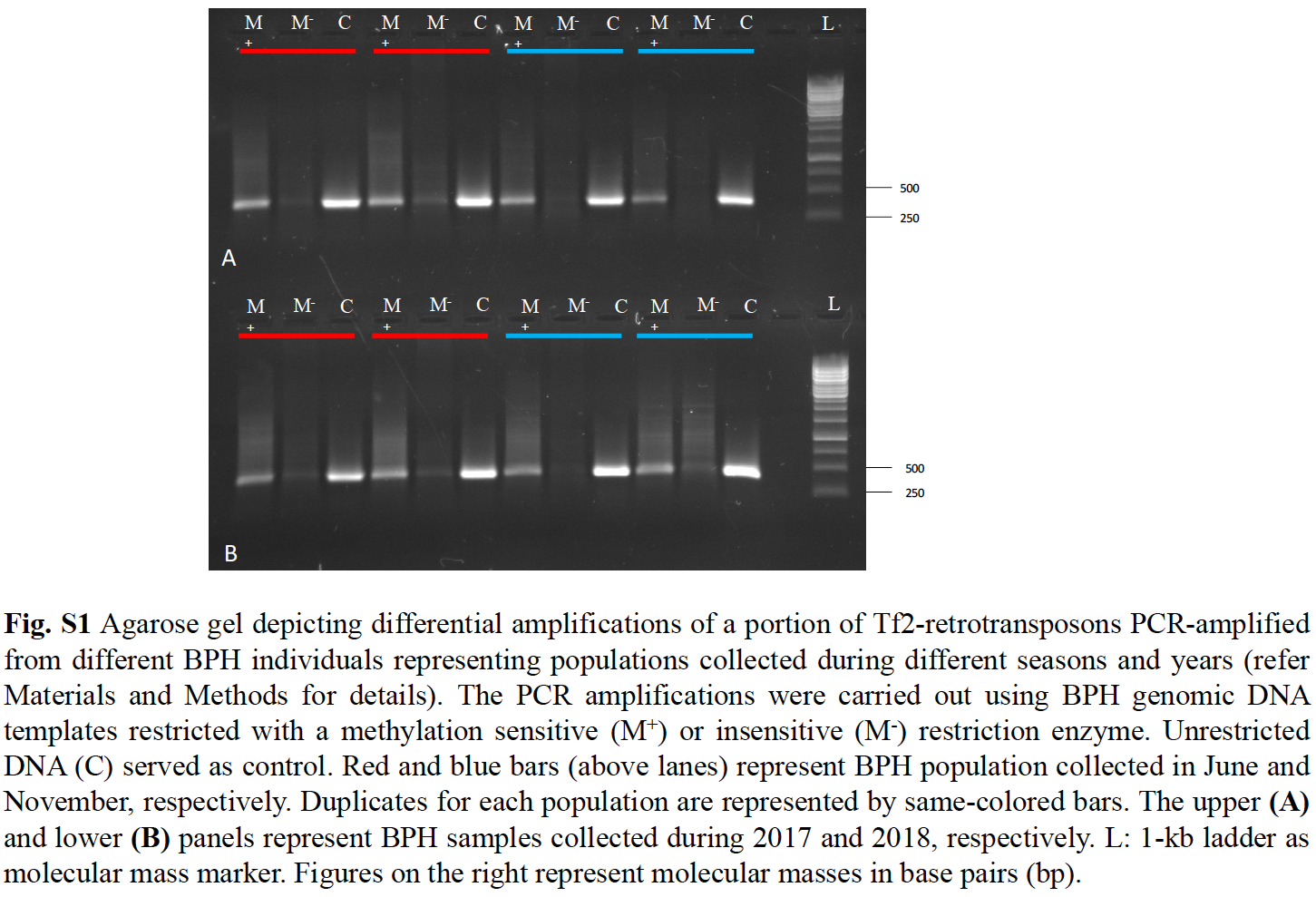


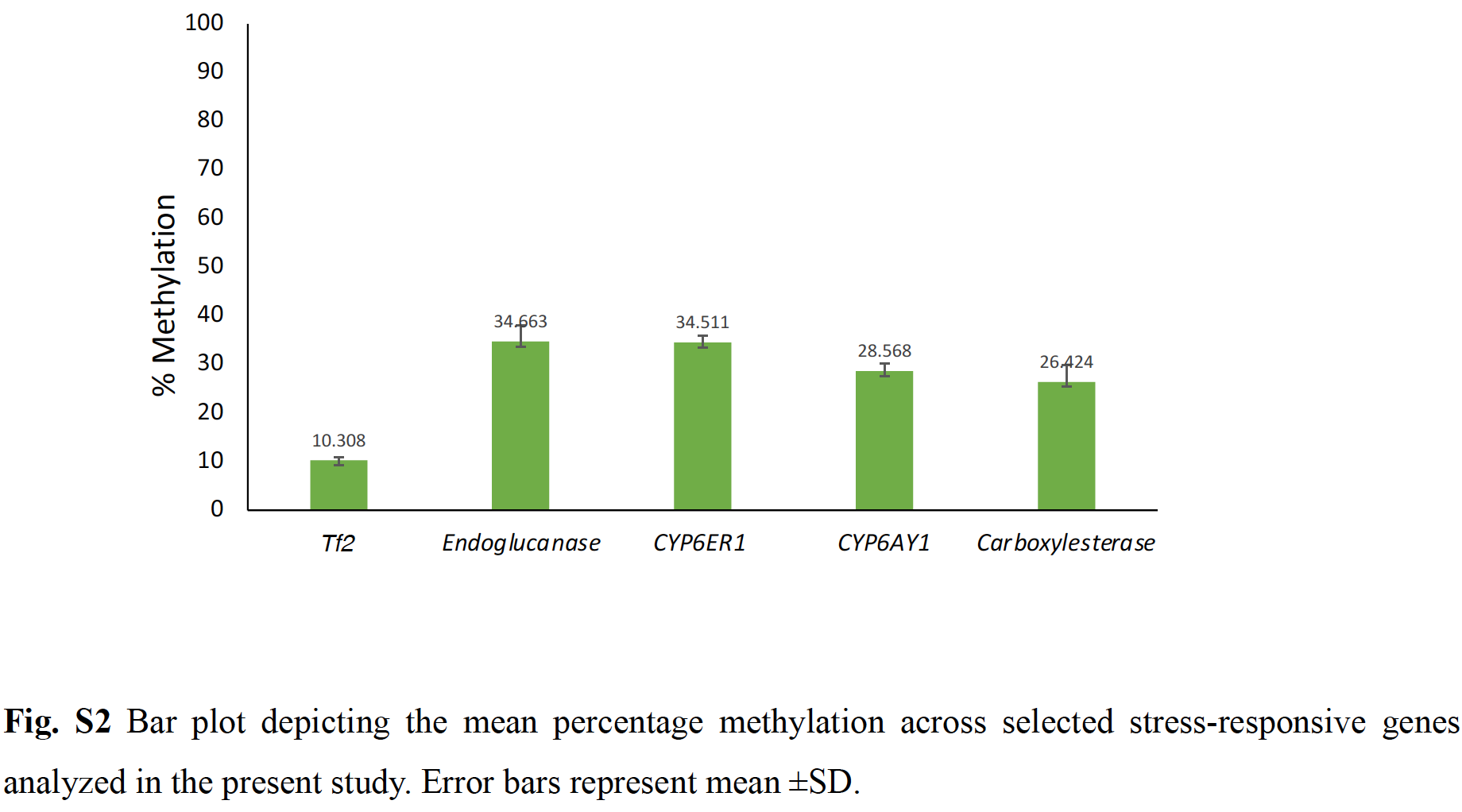


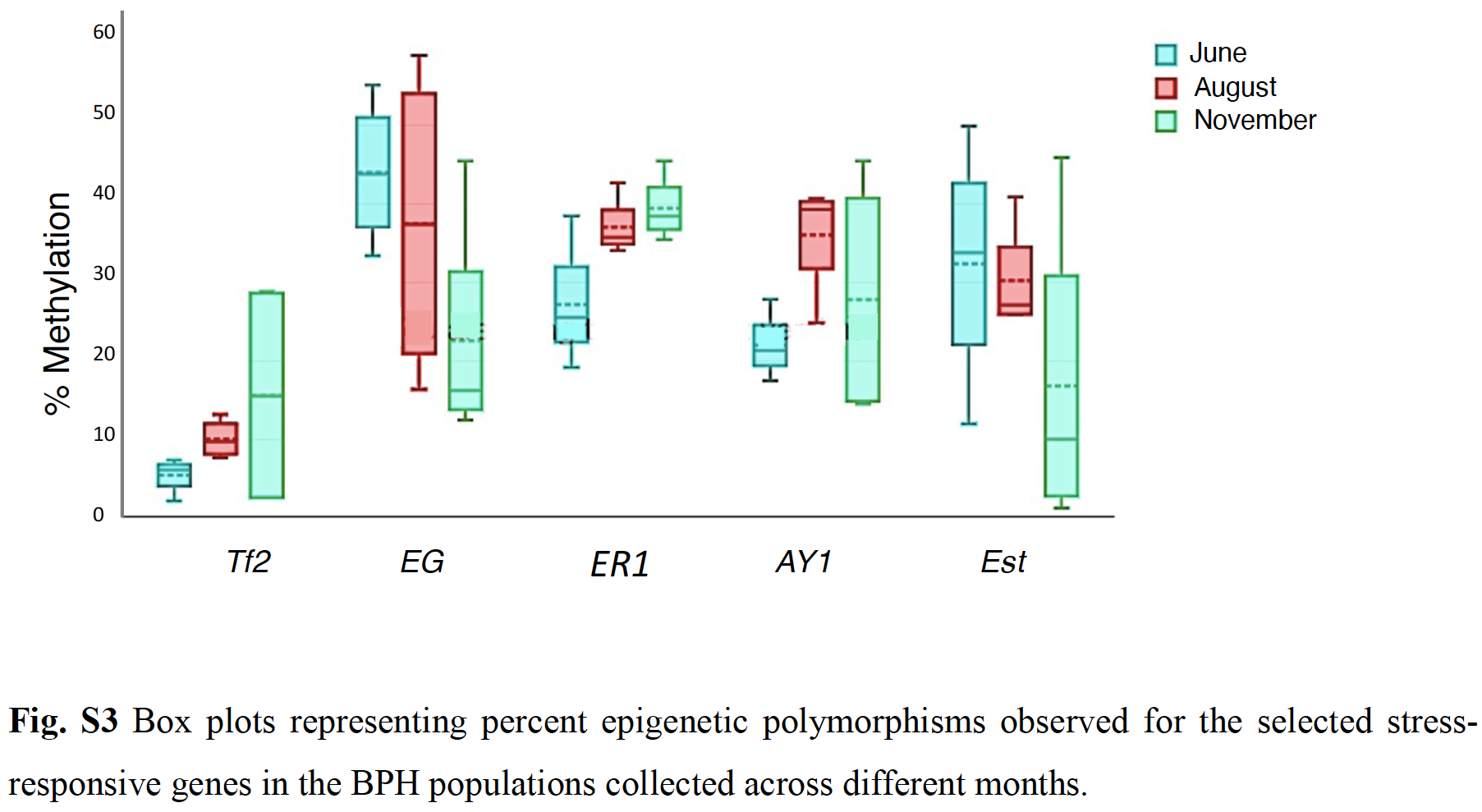


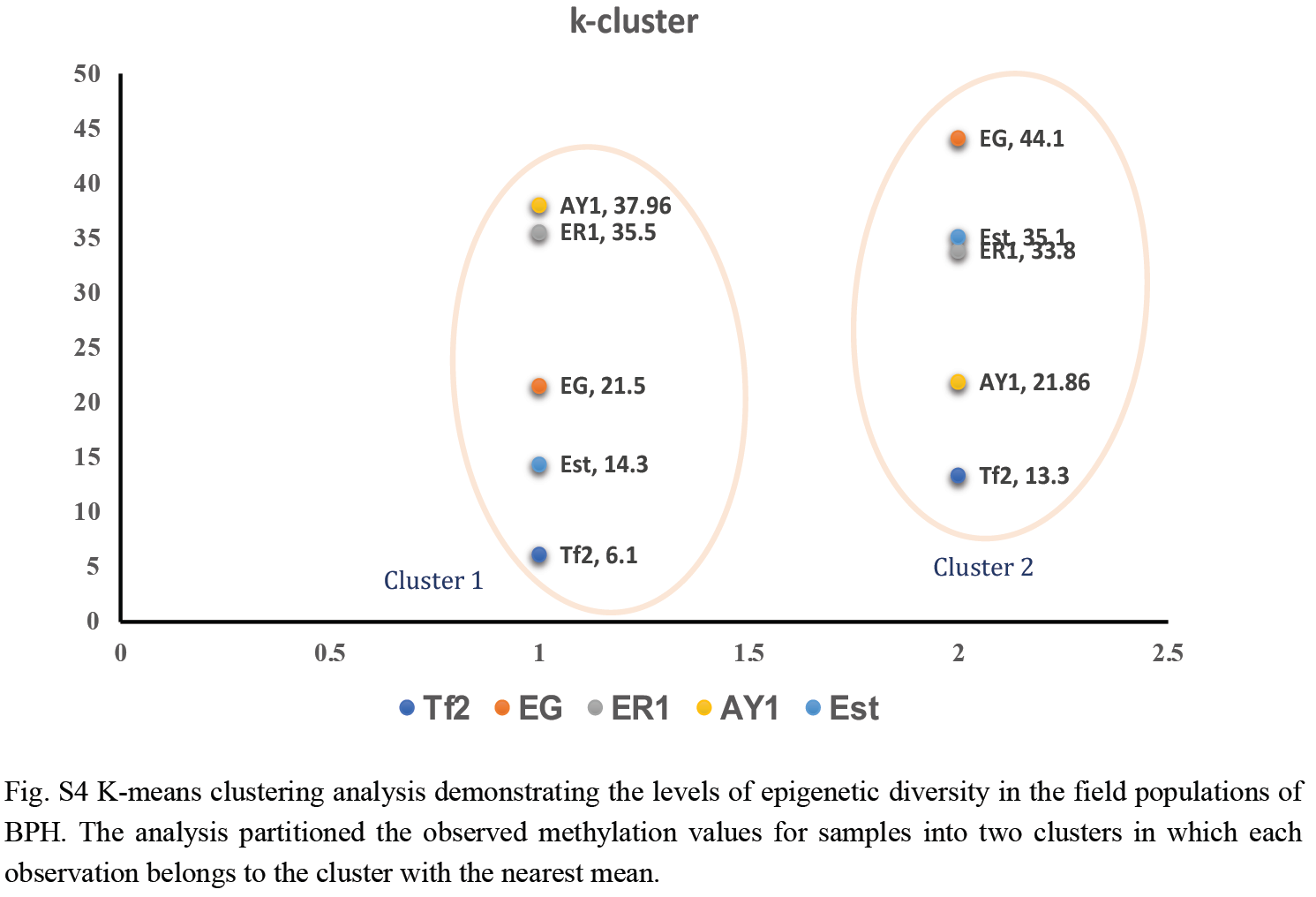


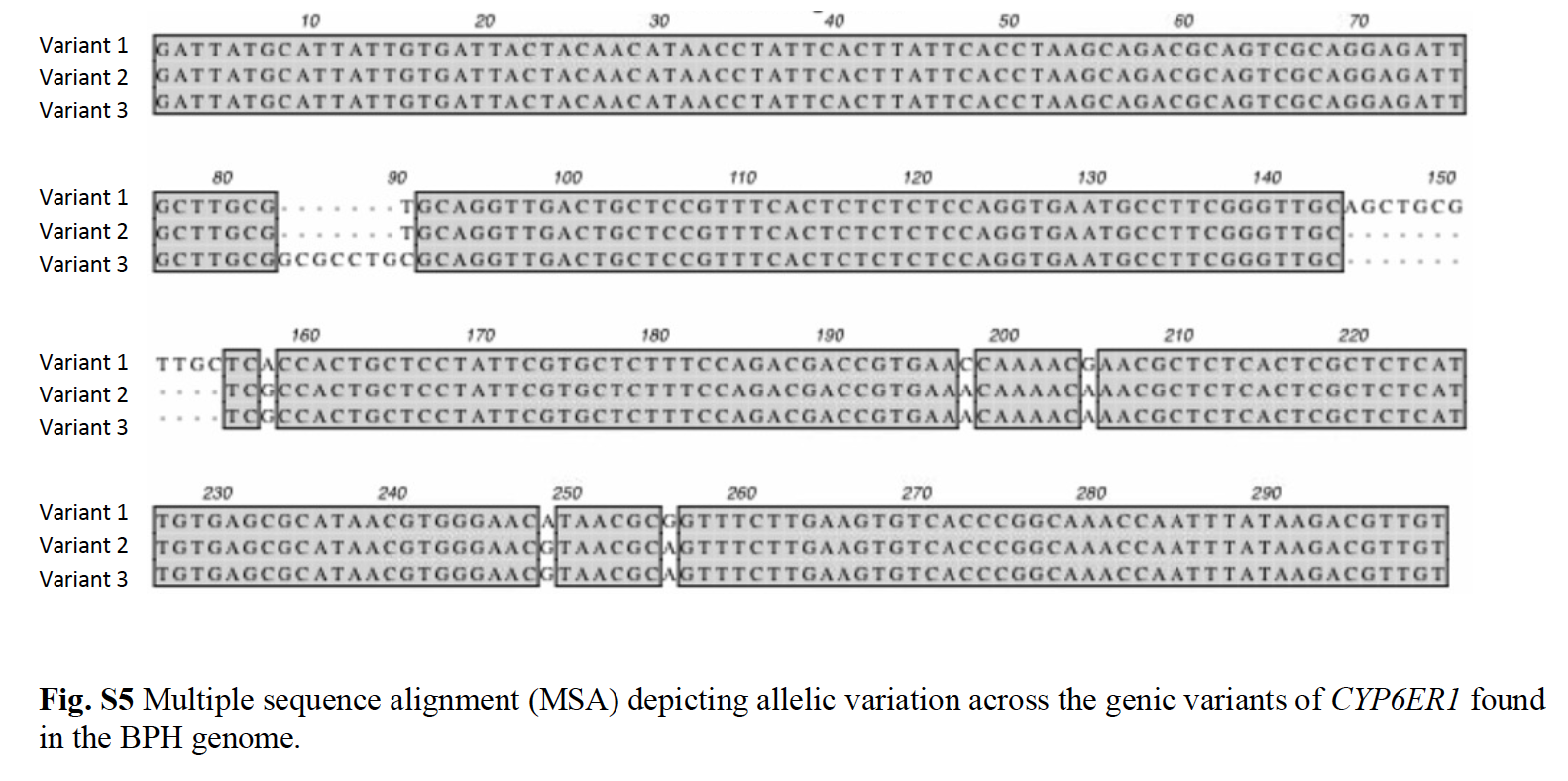


**Table S1. PCR primers used in this study.**

| **Primer Name** | **Primer Sequence (5’ ­– 3’)** | **Tm** | **Product Size** |
| --- | --- | --- | --- |
| CYP6ER1-F | GATTATGCATTATTGTGATTACTACA | 50^ᵒ^C | 299 bp |
| CYP6ER1-R | ACAACGTCTTATAAATTGGTTT | 46^ᵒ^C |  |
| CYP6AY1-F | GAGTGGATGTAACTTTGTTGTAGAG | 54^ᵒ^C | 297 bp |
| CYP6AY1-R | TTACTGTCTGGAAGAACRTAGCTATC | 54^ᵒ^C |  |
| EG-F | TGATATTGATGCGATGAGATTAT | 48^ᵒ^C | 300 bp |
| EG-R | TTGAAACAACAGGTATTCTTT | 45^ᵒ^C |  |
| Est-1F | TTCGCAAGTTTGGATGATG | 47^ᵒ^C | 278 bp |
| Est-1R | CTAACCTCCCTCCATTTCTCT | 52^ᵒ^C |  |
| Tf2-F | GAGGAGGACCAGGGAGAT | 53^ᵒ^C | 331 bp |
| Tf2-R | CAACTTCTTACCTTATCACATACA | 51^ᵒ^C |  |

**Table S2(a). One-way ANOVA test based on the methylation values obtained for BPH populations when sampled across Months.**

| **Dependent Variables** | | **Sum of Squares** | **df** | **Mean Square** | **F** | **Sig.** |
| --- | --- | --- | --- | --- | --- | --- |
| *Tf2* | Between Groups | 207.632 | 2 | 103.816 | 1.307 | .317 |
|  | Within Groups | 714.938 | 9 | 79.438 |  |  |
|  | Total | 922.569 | 11 |  |  |  |
| *Endoglucanase* | Between Groups | 968.795 | 2 | 484.398 | 2.024 | .188 |
|  | Within Groups | 2154.462 | 9 | 239.385 |  |  |
|  | Total | 3123.257 | 11 |  |  |  |
| *CYP6ER1* | Between Groups | 337.962 | 2 | 168.981 | 5.168 | .032^*^ |
|  | Within Groups | 294.271 | 9 | 32.697 |  |  |
|  | Total | 632.232 | 11 |  |  |  |
| *CYP6AY1* | Between Groups | 399.006 | 2 | 199.503 | 1.909 | .204 |
|  | Within Groups | 940.659 | 9 | 104.518 |  |  |
|  | Total | 1339.665 | 11 |  |  |  |
| *Carboxylesterase* | Between Groups | 570.859 | 2 | 285.429 | 1.201 | .345 |
|  | Within Groups | 2138.980 | 9 | 237.664 |  |  |
|  | Total | 2709.838 | 11 |  |  |  |

*The mean difference is significant at the p < 0.05

**Table S2(b). One-way ANOVA test based on the methylation values obtained for BPH populations when sampled across Years.**

| **Dependent Variables** | | **Sum of Squares** | **df** | **Mean Square** | **F** | **Sig.** |
| --- | --- | --- | --- | --- | --- | --- |
| *Tf*2 | Between Groups | 285.187 | 1 | 285.187 | 4.474 | .061 |
|  | Within Groups | 637.382 | 10 | 63.738 |  |  |
|  | Total | 922.569 | 11 |  |  |  |
| *Endoglucanase* | Between Groups | 963.021 | 1 | 963.021 | 4.458 | .061 |
|  | Within Groups | 2160.236 | 10 | 216.024 |  |  |
|  | Total | 3123.257 | 11 |  |  |  |
| *CYP6ER1* | Between Groups | 37.701 | 1 | 37.701 | .634 | .444 |
|  | Within Groups | 594.531 | 10 | 59.453 |  |  |
|  | Total | 632.232 | 11 |  |  |  |
| *CYP6AY1* | Between Groups | 427.452 | 1 | 427.452 | 4.686 | .056 |
|  | Within Groups | 912.213 | 10 | 91.221 |  |  |
|  | Total | 1339.665 | 11 |  |  |  |
| *Carboxylesterase* | Between Groups | 1011.820 | 1 | 1011.820 | 5.959 | .035^*^ |
|  | Within Groups | 1698.019 | 10 | 169.802 |  |  |
|  | Total | 2709.838 | 11 |  |  |  |

*The mean difference is significant at the p < 0.05

**Table S3. K-means clustering analysis^1^ demonstrating the levels of epigenetic diversity with regard to the methylation status in the field populations of BPH**

| **Genes** | **Cluster 1** | **Cluster 2** |
| --- | --- | --- |
| *Tf2* | 6.10 | 13.30 |
| *EG* | 21.50 | 44.10 |
| *ER1* | 35.50 | 33.80 |
| *AY1* | 37.96 | 21.86 |
| *Est* | 14.30 | 35.10 |

^1^This analysis partitioned the observed methylation values for samples across months and years into k clusters in which each observation belongs to the cluster with the nearest mean.

**Table S4. Post hoc tests performed to unravel true differences between populations (Method: Fisher’s least significant difference; LSD).**

| **Dependent Variable** | **(I) MONTH, Period 12** | **(J) MONTH, Period 12** | **Mean Difference (I-J)** | **Std. Error** | **Sig.** | **95% Confidence Interval** | **95% Confidence Interval** |
| --- | --- | --- | --- | --- | --- | --- | --- |
|  |  |  |  |  |  | **Lower Bound** | **Upper Bound** |
| *Tf2* | 6 | 8 | -4.625^*^ | 1.506 | .022 | -8.311 | -.939 |
|  |  | 11 | -10.175^*^ | 1.506 | .001 | -13.861 | -6.489 |
|  | 8 | 6 | 4.625^*^ | 1.506 | .022 | .939 | 8.311 |
|  |  | 11 | -5.550^*^ | 1.506 | .010 | -9.236 | -1.864 |
|  | 11 | 6 | 10.175^*^ | 1.506 | .001 | 6.489 | 13.861 |
|  |  | 8 | 5.550^*^ | 1.506 | .010 | 1.864 | 9.236 |
| *Endoglucanase* | 6 | 8 | 6.510 | 8.322 | .464 | -13.854 | 26.874 |
|  |  | 11 | 21.463^*^ | 8.322 | .042 | 1.099 | 41.826 |
|  | 8 | 6 | -6.510 | 8.322 | .464 | -26.874 | 13.854 |
|  |  | 11 | 14.953 | 8.322 | .123 | -5.411 | 35.316 |
|  | 11 | 6 | -21.463^*^ | 8.322 | .042 | -41.826 | -1.099 |
|  |  | 8 | -14.953 | 8.322 | .123 | -35.316 | 5.411 |
| *CYP6ER1* | 6 | 8 | -9.863^*^ | 3.571 | .033 | -18.601 | -1.124 |
|  |  | 11 | -12.265^*^ | 3.571 | .014 | -21.004 | -3.526 |
|  | 8 | 6 | 9.863^*^ | 3.571 | .033 | 1.124 | 18.601 |
|  |  | 11 | -2.402 | 3.571 | .526 | -11.141 | 6.336 |
|  | 11 | 6 | 12.265^*^ | 3.571 | .014 | 3.526 | 21.004 |
|  |  | 8 | 2.402 | 3.571 | .526 | -6.336 | 11.141 |
| *CYP6AY1* | 6 | 8 | -14.0550^*^ | 4.029 | .013 | -23.9154 | -4.1946 |
|  |  | 11 | -5.8150 | 4.029 | .199 | -15.6754 | 4.0454 |
|  | 8 | 6 | 14.0550^*^ | 4.029 | .013 | 4.1946 | 23.9154 |
|  |  | 11 | 8.2400 | 4.029 | .087 | -1.6204 | 18.1004 |
|  | 11 | 6 | 5.8150 | 4.029 | .199 | -4.0454 | 15.6754 |
|  |  | 8 | -8.2400 | 4.029 | .087 | -18.1004 | 1.6204 |
| *Carboxylesterase* | 6 | 8 | 2.125 | 8.583 | .813 | -18.879 | 23.129 |
|  |  | 11 | 15.578 | 8.583 | .119 | -5.426 | 36.581 |
|  | 8 | 6 | -2.125 | 8.583 | .813 | -23.129 | 18.879 |
|  |  | 11 | 13.453 | 8.583 | .168 | -7.551 | 34.456 |
|  | 11 | 6 | -15.578 | 8.583 | .119 | -36.581 | 5.426 |
|  |  | 8 | -13.453 | 8.583 | .168 | -34.456 | 7.551 |

Based on observed means

The error term is Mean Square (Error) = 147.363

*The mean difference is significant at the p < 0.05

**Table S5. Pairwise correlation analysis depicting inter-relatedness of genes with regard to their methylation status in field populations of BPH.**

| **Variable** | **-** | ***Tf2*** | | ***EG*** | | ***ER1*** | | ***AY1*** | ***Est*** | | |
| --- | --- | --- | --- | --- | --- | --- | --- | --- | --- | --- | --- |
| 1. *Tf2* | Pearson's r | — |  |  |  |  |  |  |  |  |  |
|  | p-value | — |  |  |  |  |  |  |  |  |  |
|  | Spearman's rho | — |  |  |  |  |  |  |  |  |  |
|  | p-value | — |  |  |  |  |  |  |  |  |  |
|  | Kendall's Tau B | — |  |  |  |  |  |  |  |  |  |
|  | p-value | — |  |  |  |  |  |  |  |  |  |
| 2. *EG* | Pearson's r | -0.510 |  | — |  |  |  |  |  |  |  |
|  | p-value | 0.109 |  | — |  |  |  |  |  |  |  |
|  | Spearman's rho | -0.422 |  | — |  |  |  |  |  |  |  |
|  | p-value | 0.196 |  | — |  |  |  |  |  |  |  |
|  | Kendall's Tau B | -0.249 |  | — |  |  |  |  |  |  |  |
|  | p-value | 0.287 |  | — |  |  |  |  |  |  |  |
| 3. *ER1* | Pearson's r | 0.531 |  | -0.597 |  | — |  |  |  |  |  |
|  | p-value | 0.093 |  | 0.052 |  | — |  |  |  |  |  |
|  | Spearman's rho | 0.641 | * | -0.619 | * | — |  |  |  |  |  |
|  | p-value | 0.034 |  | 0.042 |  | — |  |  |  |  |  |
|  | Kendall's Tau B | 0.466 | * | -0.341 |  | — |  |  |  |  |  |
|  | p-value | 0.046 |  | 0.145 |  | — |  |  |  |  |  |
| 4. *AY1* | Pearson's r | -0.324 |  | -0.188 |  | 0.405 |  | — |  |  |  |
|  | p-value | 0.332 |  | 0.580 |  | 0.216 |  | — |  |  |  |
|  | Spearman's rho | 0.071 |  | -0.039 |  | 0.073 |  | — |  |  |  |
|  | p-value | 0.835 |  | 0.909 |  | 0.831 |  | — |  |  |  |
|  | Kendall's Tau B | 0.133 |  | -0.071 |  | 0.072 |  | — |  |  |  |
|  | p-value | 0.570 |  | 0.762 |  | 0.757 |  | — |  |  |  |
| 5. *Est* | Pearson's r | -0.077 |  | 0.149 |  | -0.471 |  | -0.304 |  | — |  |
|  | p-value | 0.821 |  | 0.663 |  | 0.144 |  | 0.364 |  | — |  |
|  | Spearman's rho | -0.034 |  | 0.140 |  | -0.378 |  | -0.249 |  | — |  |
|  | p-value | 0.922 |  | 0.681 |  | 0.252 |  | 0.461 |  | — |  |
|  | Kendall's Tau B | -0.018 |  | 0.148 |  | -0.248 |  | -0.159 |  | — |  |
|  | p-value | 0.939 |  | 0.525 |  | 0.288 |  | 0.495 |  | — |  |

Conditioned on variables: YEAR_ MONTH

*significant at p < 0.05

**Table S6. Stress-responsive genes analysed in the present study along with the number of predicted CpG islands, the restriction enzyme combination used to detect site-specific methylation and the size of the CpG island of each gene assayed along with the primer (see Table S1) binding co-ordinates.**

| **Gene** | **Accession Number** | **Number of CpG Islands** | **CpG Island Location** | **Primer Binding Region** | **Length of CpG Island (bp)** | **Methylation Sensitive/Insensitive RE*** **Combination** | **No. of Restriction Sites** |
| --- | --- | --- | --- | --- | --- | --- | --- |
| *Tf2* | XM_022332395.2 | 1 | Exonic | 40-392 | 353 | HpaII/MspI | 4 |
| *EG* | LOC111056901 | 1 | Intronic | 4281-4581 | 300 | HpaII/MspI | 1 |
| *CYP6ER1* | MF970460.1 | 2 | Exonic | 10321- 10619 | 299 | HpaII/MspI | 1 |
| *CYP6AY1* | LOC111048315 | 1 | Exonic | 7026-7323 | 297 | AatII/FatI | 2 |
| *Est* | LOC111062288 | 1 | Exonic | 6985-7218 | 233 | BsmAI/BtsCI | 1 |

*RE: Restriction Enzyme
